# Dr. Dock’s Article

**Published:** 1891-04

**Authors:** 


DR. DOCK'S ARTICLE will be read with much interest, not only in America, but in Europe, it being, we believe, the fourth report of original research upon the *amœba coli*, published in this country. If the doctor has not demonstrated the relation which always exists between this organism and the disease, he has at least pointed to a strong presumption of causation, in a large number of cases. Dr. Dock is an enthusiastic and very skillful worker in the field of pathology and bacteriology, and has doubtless a brilliant career opening up to him.

